# Multi-Factor Regulation of the Master Modulator LeuO for the Cyclic-(Phe-Pro) Signaling Pathway in *Vibrio vulnificus*

**DOI:** 10.1038/s41598-019-56855-4

**Published:** 2019-12-27

**Authors:** Na-Young Park, In Hwang Kim, Yancheng Wen, Keun-Woo Lee, Sora Lee, Jeong-A Kim, Kwang-Hwan Jung, Kyu-Ho Lee, Kun-Soo Kim

**Affiliations:** 1 Department of Life Science, Sogang University, Seoul, Korea; 2 Interdisciplinary Program of Integrated Biotechnology, Sogang University, Seoul, Korea; 3 Institute of Biological Interfaces3, Sogang University, Seoul, Korea; 4 Key Laboratory of Ministry of Education for Gastrointestinal Cancer Research Center for Molecular Medicine, Fujian Medical University, Fuzhou, Fujian, People’s Republic of China

**Keywords:** Bacterial genes, Pathogens

## Abstract

LeuO plays the role of a master regulator in the cyclic-L-phenylalanine-L-proline (cFP)-dependent signaling pathway in *Vibrio vulnificus*. cFP, as shown through isothermal titration calorimetry analysis, binds specifically to the periplasmic domain of ToxR. Binding of cFP triggers a change in the cytoplasmic domain of ToxR, which then activates transcription of *leuO* encoding a LysR-type regulator. LeuO binds to the region upstream of its own coding sequence, inhibiting its own transcription and maintaining a controlled level of expression. A five-bp deletion in this region abolished expression of LeuO, but a ten-bp deletion did not, suggesting that a DNA bending mechanism is involved in the regulation. Furthermore, binding of RNA polymerase was significantly lower both in the deletion of the ToxR binding site and in the five-bp deletion, but not in the ten-bp deletion, as shown in pull-down assays using an antibody against RNA polymerase subunit α. In summary, multiple factors are involved in control of the expression of LeuO, a master regulator that orchestrates downstream regulators to modulate factors required for survival and pathogenicity of the pathogen.

## Introduction

*Vibrio vulnificus* is a gram negative, motile, curved bacterium^1^. It is an opportunistic human pathogen that causes septicemia and leads to high mortality^1^. This pathogenic *Vibrio* species produces a diketopiperazine (DKP) compound called cyclic-phenylalanine proline (cFP), that reaches maximum levels when cells enter stationary phase and triggers the expression of a series of genes associated with pathogenicity^2^. This compound has been identified in the related pathogenic species *V. cholerae* and *V. parahaemolyticus*, as well as the non-pathogen *V. harveyi*^2,3^, suggesting that it is a common signal molecule among the Vibrionaceae. Based on these findings, cFP was proposed to be a novel quorum-sensing signal molecule^2,4^. However, cFP signaling has been reported to have a variety of biological activities including: antibacterial and antifungal activities^5,6^, induction of an intestinal-like differentiation of human colon carcinoma cells^7^, inhibition of DNA topoisomerase^8^ and cancer cell growth, and induction of apoptosis in colon cancer cells^9^. cFP also inhibits interferon (IFN)-β production by inducing a conformational change in retinoic-acid-inducible gene-I (RIG-I)^10^, affects innate immune responses^11^, and causes apoptosis of human cell lines by elevating intracellular levels of reactive oxygen species (ROS)^12^. These results suggest that cFP is not only a signaling molecule but also a virulence factor.

In *V. vulnificus* and *V. cholerae*, the cFP signal is transduced via the inner membrane protein ToxR^2,13^, which is known to form either homodimers or heterodimers with ToxS^14^. ToxR activates the ToxT regulon in *V. cholerae*, which includes genes encoding CT (cholera toxin), TCP (the toxin-coregulated pilus), and accessory colonization factor (ACF)^15–17^. ToxR, independent of ToxT, also activates *ompU* expression and represses *ompT* expression^18–20^. cFP interacts with ToxRS to elevate levels of LeuO, which consequently represses transcription of *aphA*, decreasing the expression of CT and TCP^2,3,13^. Recently, it was reported that cFP increases the expression of catalase, which in turn aids in the survival of *V. vulnificus* in the host environment where ROS levels are themselves enhanced by the action of cFP from the pathogen^21^. The cFP signal is transduced from ToxR to LeuO, which subsequently activates the expression of the histone-like proteins vHUα and vHUβ. These proteins lead to increased intracellular levels of the alternative sigma factor RpoS, which is well known to be associated with stress responses, by stabilizing the transcript^22^. Expression of catalase is dependent on RpoS. Given the fact that LeuO, vHUα and vHUβ, and RpoS are each responsible for the regulation of separate regulons, and that many of the target genes are associated with pathogenicity, it is clear that LeuO is a key regulatory component of the cFP-signaling network, as is the case for the human pathogen *V. cholerae*.

LeuO is a member of the LysR-type transcriptional regulator (LTTR) family, which is a well-characterized group of transcriptional regulators^23^. Members of this family have been identified in many bacteria, including enteric pathogens such as *Escherichia coli, Salmonella enterica* serovar *Typhimurium, Enterobacter cloacae*, and *Vibrio* spp^23^. LeuO regulates a wide variety of genes that are involved in amino acid biosynthesis, catabolism of aromatic compounds, antibiotic resistance, nitrogen fixation, oxidative stress response, quorum sensing, and virulence^24–27^. The structure of LTTR proteins includes an N-terminal DNA-binding helix-turn-helix motif and a C-terminal co-inducer-binding domain^23,28^. The DNA binding motif for this family is ambiguous but generally consists of AT-rich sequences^23^.

This study identified elements responsible for the regulation of LeuO in the human pathogen *V. vulnificus*, pointing to its role as a master regulator for the cFP-dependent signaling pathway and elucidating the underlying mechanisms for these elements, highlighting the complexity and importance of this pathway.

## Results

### cFP induces the expression of LeuO in a ToxR-dependent manner

**Figure 1:**
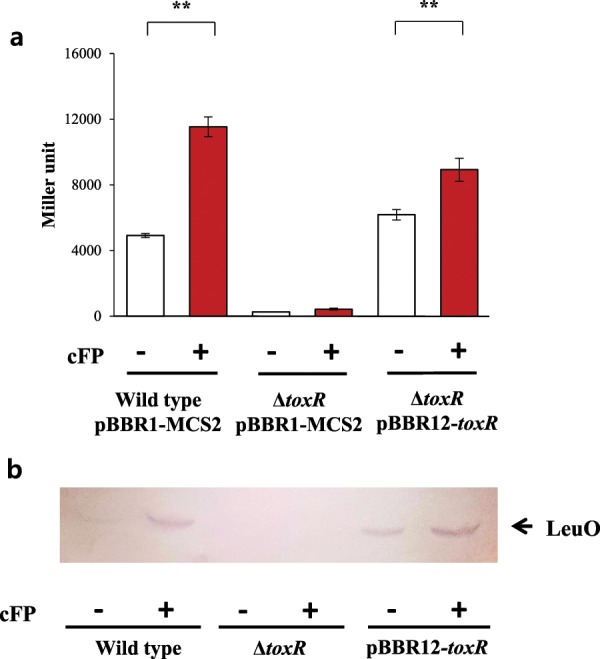
cFP induces the expression of LeuO via the membrane sensor protein ToxR. (**a**) β-Galactosidase activity of a *leuO*-*lacZ* fusion in the absence (open bars) and presence (solid bars) of exogenous 5 mM cFP in wild type *V*. *vulnificus*, a *toxR*-deletion isotype, and the deletion mutant supplemented with *toxR in trans* (pBBR12-*toxR*). The error bars denote standard deviations of the results of three independent experiments (**P < 0.005). (**b**) Western hybridization using a polyclonal antibody against LeuO of total proteins from wild type *V*. *vulnificus*, *toxR*-deletion, and the *toxR*-deletion mutant supplemented with *toxR in trans* (pBBR12-*toxR*) grown in the absence and the presence of cFP.

**Figure 2:**
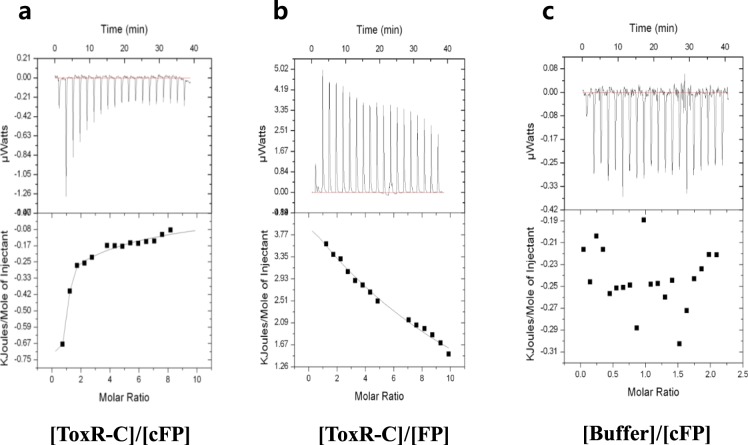
Isothermal titration calorimetric analysis shows binding between the periplasmic domain of ToxR (ToxR-C) and cFP. The change in heat capacity was measured for: (**a**) purified ToxR-C (0.2 mM) with increasing amounts of cFP, (**b**) purified ToxR-C (0.2 mM) with increasing amounts of the linear dipeptide phenylalanine-proline, or (**c**) increasing amounts of cFP in the absence of ToxR-C.

We showed previously, both through DNA microarray analysis^29^ and next-generation sequencing of total mRNA^30^, that a gene encoding a homolog of the cytoplasmic transcriptional regulator LeuO (GenBank VVM06_02645) was induced by cFP in *V*. *vulnificus*. Quantitative measurements of *leuO* expression using a *lacZ*-transcriptional fusion indicated that cFP induced the expression of *leuO* about three-fold in wild type *V*. *vulnificus* strain MO6-24/O, but not in a *toxR*-deletion mutant (Fig. 1a). Re-introduction of *toxR in trans* on a plasmid into the mutant restored the cFP-dependent induction of *leuO*. We then employed western hybridization using an antibody against LeuO, and observed the same result at the protein level. In a wild type strain, LeuO was expressed at higher levels in the presence of cFP, while in the absence of cFP or in a *toxR*-deletion mutant with or without cFP, there was no detectable LeuO (Fig. 1b). When *toxR* was re-introduced *in trans* on a plasmid, the expression of LeuO is restored to the wild type at the protein level. From these results, we concluded that *leuO* is induced by cFP in a ToxR-dependent manner. It has been proposed that the C-terminal periplasmic domain of ToxR acts as a sensor of environmental stimuli^14^, therefore we predicted that this is also the domain to which cFP binds. To test this prediction, a His-tagged version of the C-terminal domain of ToxR (ToxR-C) was overexpressed in *E. coli* and purified. Isothermal titration calorimetry (ITC) was employed to measure the binding parameters of 0.2 mM of the purified ToxR-C in combination with increasing amounts of cFP. The results, as shown Fig. 2a, suggest that ToxR-C bound to cFP at a ratio of approximately 1:1, with a *K*_*d*_ of 4.2 × 10^−5^ ± 0.26 × 10^−5^ M. In contrast, the linear dipeptide phenylalanyl proline, used as a control, showed nonspecific binding to ToxR-C (Fig. 2b) and no binding signal was detected in the presence of the reference buffer alone (Fig. 2c). These results indicated that cFP does indeed bind specifically to the periplasmic domain of ToxR.

### The N-terminal domain of ToxR binds to a *cis*-element upstream of *leuO*

**Figure 3:**
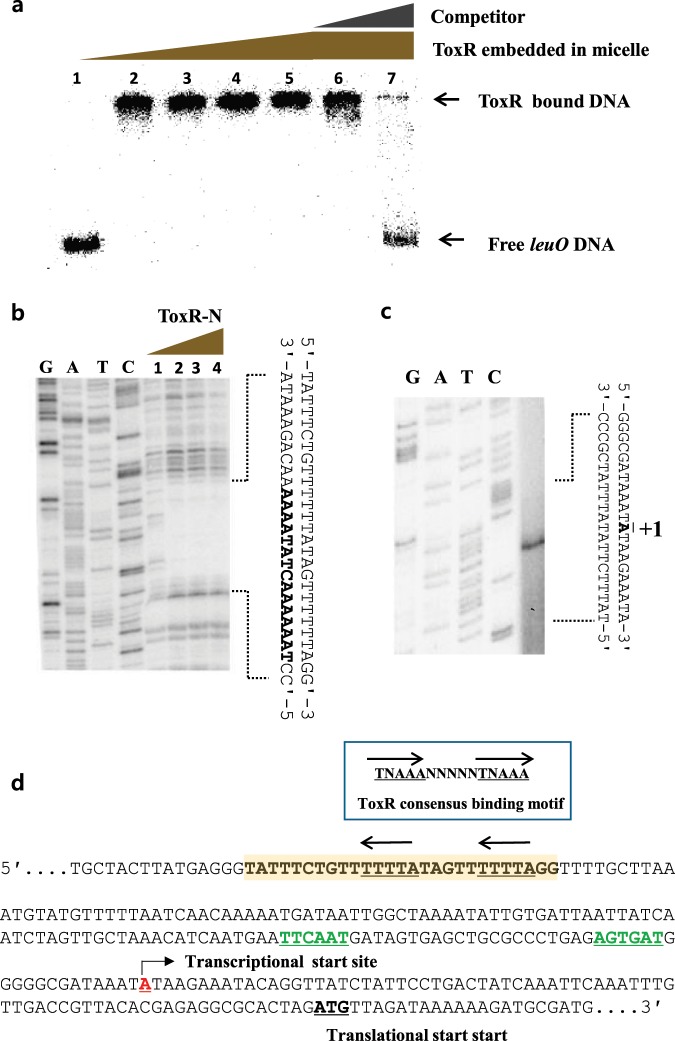
Analysis of the ToxR-binding region and transcription start site in the DNA region upstream of *leuO*. (**a**) Gel-mobility shift assay of a labeled 412-bp DNA fragment from the *leuO* promoter region using purified ToxR. Lanes 1-5: ToxR at concentrations of 0, 200, 400, 600, and 800 nM. Lanes 6-7: 800 nM ToxR with 10, and 100 ng of the same DNA unlabeled. (**b**) DNaseI protection assay of the region upstream of *leuO* using ToxR-N. Lanes 1-4: ToxR-N at 0, 1, 2, and 4 μM incubated with 200 ng of labeled *leuO* promoter DNA and separated by gel electrophoresis alongside a sequencing ladder (left four lanes). The sequence that matched the consensus ToxR binding site is shown in bold text. (**c**) Localization of the *leuO* transcription start site by primer extension. A primer complementary to the 5′-end of the gene was used, as described Materials and Methods. (**d**) Genetic map of the DNA region upstream of *leuO*. The ToxR binding sequences are highlighted in yellow and the consensus ToxR binding sequences^32^ are shown in the box above. The promoter and transcriptional start site are underlined in green and red text, respectively, and the translational start site is bolded and underlined.

We predicted that ToxR induces the expression of *leuO* by binding to a *cis*-element upstream of *leuO* as it does for *ompU* in *V. cholerae*^18^. ToxR is an inner membrane protein that has both an N-terminal cytoplasmic domain (ToxR-N) with a DNA binding motif^31^ and a C-terminal periplasmic domain (ToxR-C). To measure ToxR binding upstream of *leuO*, we prepared purified ToxR in detergent (see Methods) and performed gel-mobility shift assays. As shown in Fig. 3a, ToxR bound to this DNA fragment, and non-labeled DNA outcompeted the labeled DNA, showing that the protein specifically binds to the region. Footprinting analysis using ToxR-N pointed to a specific binding site between −229 and −215 with respect to the start codon (Fig. 3b), and defined the binding site as ‘TAAAAAACTATAAAAAACAGAAATA’ (underlined sequences coincide with the known consensus ToxR binding site; 5′-TNAAA-N_5_-TNAAA-3′ in *V*. *cholerae*^32^). We also defined the transcriptional start site of *leuO* as 73 bases upstream of the translational start site and identified putative −35 and −10 promoter regions that have a low similarity to the canonical promoter consensus sequences (Fig. 3c). The location of each these sites in the *leuO* DNA sequence are shown in Fig. 3d.

### cFP enhances the affinity of ToxR binding to the *cis*-acting element of *leuO*

**Figure 4:**
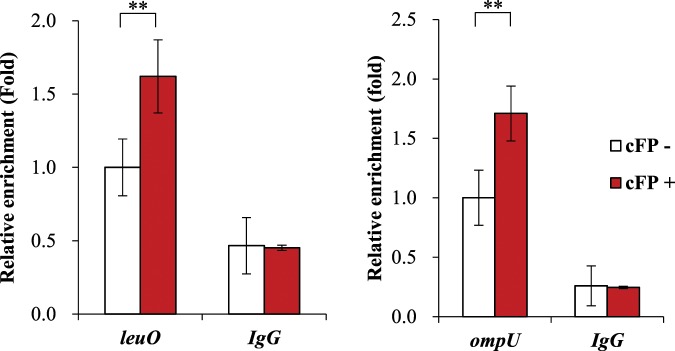
ChIP analysis showing that cFP enhances the ToxR binding affinity for the *cis*-acting elements in the DNA region upstream of *leuO* and *ompU*. The *toxR* null mutant cells expressing strep-tagged ToxR were cross-linked, washed, and sonicated as described in Materials and Methods. Lysates were then treated with either anti-Strep-tag II monoclonal antibody or normal mouse IgG as a control. Free DNA was purified and analyzed by quantitative real-time PCR (qRT-PCR) on a Light Cycler 480 II real-time PCR system. The relative enrichment was calculated as the amount of transcript compared to transcript from cells without cFP. Values are averages from biological experiments done in triplicate. Error bars indicate the standard deviations (**P < 0.005).

Knowing that cFP binds to the C-terminal periplasmic domain of ToxR and that the N-terminal cytoplasmic domain of ToxR binds upstream of *leuO* led to the hypothesis that cFP is a signaling molecule that drives intracellular expression of target genes such as *leuO*. To test this hypothesis, we employed chromatin immunoprecipitation (ChIP) analysis^33^ using an antibody against ToxR, and observed a higher level of binding of ToxR to the *leuO* upstream DNA in the presence of cFP than in the absence of cFP (Fig. 4). The same effect was observed for *ompU*, a gene known to be induced by cFP-ToxR signaling^2^, and no effect was seen for the negative control, a non-specific IgG.

### LeuO represses transcription of its own coding gene

**Figure 5:**
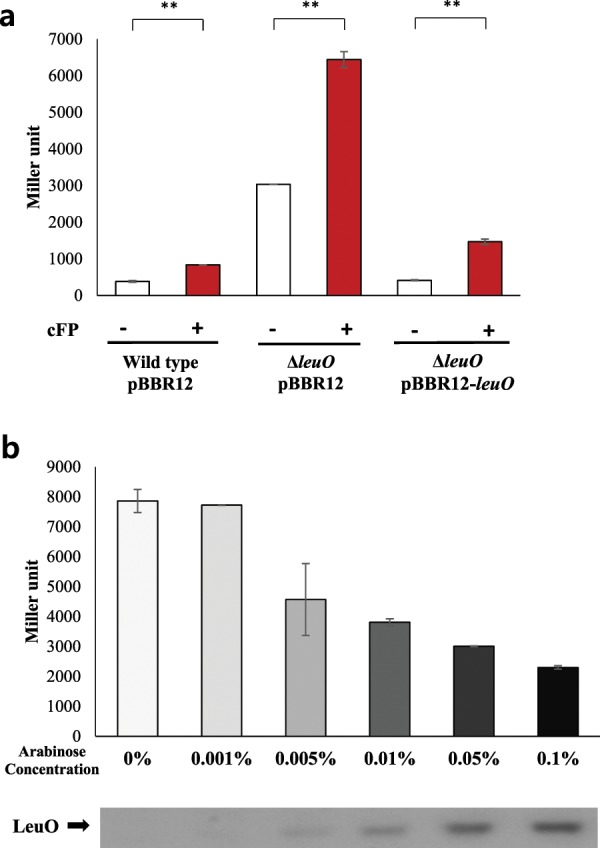
LeuO regulates the transcription of its own coding gene. (**a**) β-Galactosidase activity of a *leuO*-*lacZ* fusion in the absence (open bars) and presence (solid bars) of exogenous 5 mM cFP in wild type *V*. *vulnificus*, a *leuO*-deletion isotype, and the deletion mutant with *leuO in trans* (pBBR12-*leuO*). The error bars denote standard deviations of three independent experiments (**P < 0.005). (**b**) Transcription levels of *leuO* as measured by β*-*Galactosidase activities from ∆*leuO* (pBBR12-leuO-ara) harboring pMZtc-*leuO* (upper panel), and translation levels as determined by western hybridization using an antibody against LeuO (lower panel). Culture conditions are described in Materials and Methods. To induce the expression of LeuO, the culture was split into six aliquots after reaching an A_600_ of 0.4, and then arabinose was added to each at concentrations ranging from 0–0.1%. Error bars denote standard deviations of three independent experiments.

In some organisms, LeuO has been shown to be self-regulated^23,34^. To determine whether or not this is the case in *V. vulnificus*, we introduced a *leuO*-*lacZ* transcriptional fusion vector into both wild type and *leuO*-deletion mutant cells and quantitatively measured β-galactosidase activity in the presence or absence of exogenous cFP. In the *leuO*-deletion mutant, β-galactosidase levels were much higher than that of wild type (Fig. 5a). When *leuO* was re-introduced *in trans* on another plasmid, β-galactosidase activity was nearly as low as wild type. We then measured β-galactosidase activity from a chromosomal *leuO*-*lacZ* fusion in a ∆*leuO* isotype harboring an arabinose-inducible LeuO overexpression vector (Fig. 5b). When measured by western hybridization using antibody against the expressed protein, expression of LeuO increased as concentrations of arabinose increased. As the LeuO expression level increased, β galactosidase activity from the *leuO-lacZ* fusion was gradually decreased. These results indicate that LeuO represses its own expression in *V. vulnificus*.

### LeuO binds to multiple sites in the region upstream of *leuO*

**Figure 6:**
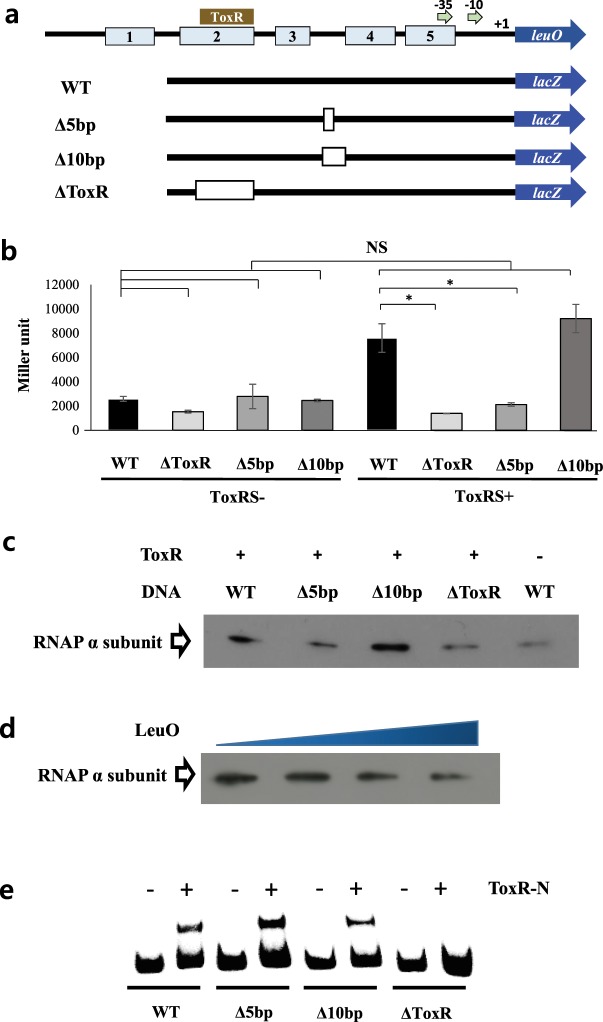
*leuO* expression is regulated by DNA bending. (**a**) Genetic map showing the upstream region of *leuO*. The binding region for ToxR, the five binding sites for LeuO (labeled 1–5), and the − 10 and − 35 promoter regions are indicated. The transcription start site (+1) also is indicated. Boxes indicate the location of the deletions. (**b**) Expression of *lacZ* fused to wild-type or mutagenized regions upstream of *leuO* in the presence and absence of exogenous ToxRS in wild type *E. coli* strain DH5α. The error bars denote standard deviations of the results of three independent experiments (*P < 0.05; NS, not significant). (**c**) *In vitro* pull-down assay. Wild-type or mutagenized upstream regions of *leuO* were incubated with RNA polymerase holoenzyme (New England Biolabs, Beverly, MA) in the presence or absence of ToxR-N, and the reactions were applied to Streptavidin Magnetic Beads (New England Biolabs, Beverly, MA). The resins were washed, and then treated with protein loading buffer as described in Materials and Methods. The precipitates were resolved by SDS-PAGE, and the amount of RNA polymerase was detected by western hybridization using an antibody against the α subunit. (**d**) *In vitro* pull-down assay. The wild type region upstream of *leuO* was incubated with RNA polymerase holoenzyme and ToxR-N with gradually increasing amounts of LeuO. Reactions were then applied to Streptavidin Magnetic Beads, washed, and treated with protein loading buffer as described in Materials and Methods. The precipitates were resolved by SDS-PAGE, and the amount of RNA polymerase was detected by western hybridization using an antibody against the α subunit. (**e**) Gel-mobility shift assay of a labeled wild-type or mutagenized upstream regions of the *leuO* promoter region using purified ToxR-N. Lanes 1, 3, 5, 7: no protein. Lanes 2, 4, 6, 8: 100 nM of ToxR-N.

Through DNA footprinting analysis (Supplementary Fig. S1), we identified the following five LeuO binding sites in the region upstream of *leuO* (with respect to the translational start site): −274 to −253 (LeuO-1), −244 to −207 (LeuO-2), −196 to −187 (LeuO-3), −167 to −141 (LeuO-4), and −137 to −109 (LeuO-5). A sequence alignment of these five LeuO binding sequences and other known LeuO binding sites suggests an AT-rich consensus sequences, **AT**NAA**T**T**T**N**TT**TA**AT**A**AAT** (more highly conserved bases noted in bold font) (Supplementary Fig. S2). The ToxR binding region overlaps with LeuO-2, and the putative −35 promoter region overlaps with LeuO-5 (Fig. 6a).

### Bending of DNA in the upstream region is important for *leuO* regulation

Transcription of *leuO* was measured in a series of deletions in the upstream region, including a deletion in the ToxR binding site and either a 5-bp or 10-bp deletion between the ToxR binding site and the promoter region (Fig. 6a). A transcriptional fusion between a *lacZ*-reporter and either the wild type upstream region of *leuO* or each of the three mutants was assessed in an *E. coli* strain harboring an arabinose-inducible ToxRS expression vector. As shown in Fig. 6b, in the absence of ToxRS, expression of all four reporter fusions was low without any significant differences among them. When ToxRS was induced, expression levels of the reporter containing the 10-bp deletion was equivalent to that of wild type, whereas the reporter from the 5-bp deletion was expressed at levels as low as in the absence of the ToxRS. This significant difference in expression levels between DNA sequences of different lengths suggests that a curve in the DNA structure between the ToxR-binding site and the promoter region may be important. It is possible that this bending allows ToxR to come in contact with RNA polymerase and stabilize its binding to the promoter region, but only when the two are in the correct orientation. To test this hypothesis, we examined the effect of various deletions on the binding of RNA polymerase. We co-incubated purified ToxR-N and RNA polymerase holoenzyme together with each of DNA fragments shown in Fig. 6a. The precipitates were resolved by SDS-PAGE and levels of RNA polymerase were measured by western hybridization using an antibody against the α subunit. As shown in Fig. 6c, a DNA fragment with a 10-bp deletion bound to RNA Pol as well as the wild type fragment. However, both the 5-bp deletion and the deletion in the ToxR binding site led to significantly less binding of RNA Pol. When ToxR was not expressed, significantly lower levels of RNA polymerase bound to the wild type upstream region. Addition of increasing amounts of LeuO to the wild type fragment led to a gradual decrease in the amount of RNA Pol that was bound (Fig. 6d). It is arguable that the 5-bp and 10-bp deletions may result in the failure of the ToxR binding and, consequently, RNA Pol could not be invited to the promoter. To test this possibility, we compared gel-mobility shift using purified ToxR-N among radiolabeled wild type DNA fragment of the upstream region of *leuO*, the same fragment with each of the 5-bp and 10-bp deletions as well as that with deletion in the ToxR binding site as described in the Methods section. As shown in Fig. 6e, the 5-bp or 10-bp deletion did not significantly affect the binding of ToxR-N comparing to the wild type fragment meanwhile deletion in the ToxR-binding site completely abolished the binding.

In summary, these results suggest that bending of the DNA between the ToxR binding site and the promoter assists in binding of RNA polymerase, ToxR is necessary for maximal binding, and LeuO interferes with RNA polymerase binding.

## Discussion

cFP has only recently been identified as a signaling molecule in *Vibrio* species. Even though production of this compound had been observed in *Pseudomonas spp*.^35^, the demonstration that it plays a signaling role was first shown in *V. vulnificus* and *V. cholerae*^2,3^. Species belonging to Vibrionaceae comprise well-studied model systems for the intercellular communication phenomena represented by quorum-sensing. Homoserine lactone (autoinducer-1, AI-1)^36^, (2S, 4S)-2-methyl-2,3,3,4-tetrahydroxytetrahydrofuran borate (AI-2), and 3-hydroxytridecan-4-one (Cholera autoinducer 1, CAI-1)^37^ have been intensively studied as quorum sensing signaling molecules. In *V*. *fischeri*, AI-1 binds directly to a LuxR-type activator resulting in up-regulation of a variety of target genes^38,39^. AI-2 and CAI-1 activate a two-component regulatory circuit and funnel the signal to a *V. harveyi* LuxR-type activator^40,41^, which then modulates expression of target genes. The way in which cFP transduces a signal is distinct from that of these other signaling molecules. cFP binds to ToxR, which then transduces the signal to LeuO. ToxR itself also regulates a set of genes, *toxT*, *ctxAB*, and *tcp*, all of which are located on a pathogenic island present only in *V. cholerae* but not in other species. ToxR also regulates expression of *ompU*, the gene we show to be under the control of LeuO as well (Fig. 4). LeuO modulates the expression of numerous other genes associated with a variety of functions in pathogenic *Vibrio* spp., such as reactive oxygen species resistance^21^, cationic antimicrobial peptide resistance^42^, virulent protease expression^43^, acid tolerance^44^, biofilm formation^45^, protein export^34^, and exopolysaccharide gene expression^46^. This points to LeuO as a master regulator that is induced by the signal transduction protein ToxR in response to signals including cFP.

ITC analysis showed that cFP binds to the periplasmic domain of ToxR with a molar ratio of approximately 1:1. It is likely that cFP binding leads to an allosteric change in the cytoplasmic domain of ToxR, which then has altered affinity for the cognate *cis*-elements of target genes. Alternatively, binding of cFP may affect the ability of ToxR to form either a homodimer or a heterodimer with ToxS, another membrane protein required for ToxR functional activities^47^. The *K*_*d*_ of cFP binding to ToxR was approximately 4 × 10^−5^ M. Considering that the concentration of cFP in culture supernatants of *V. vulnificus* is ~0.2 mM in early exponential phase and increases to ~0.8 mM by stationary phase^2^, it was predicted that the cell would sense cFP at an early stage of growth. However, it has been reported that induction of *ompU* occurs only once cells reach stationary phase^2^. One explanation for this disparity is that cFP has poor membrane permeability and doesn’t accumulate in the periplasm until extracellular concentrations are higher.

ToxR is absolutely required for the expression of *leuO*. ToxR binding sites are well conserved among ToxR target genes, but the relative position and arrangement of the ToxR binding sites in relation to the promoter sequences are quite variable^18,32^. Unlike other ToxR-regulated genes, the regions upstream of *leuO* in *V. vulnificus* and *V. cholerae* have long gaps (approximately 95 bases) between the ToxR binding site and the promoter (Supplementary Fig. S3). The region upstream of the *toxT* gene in *V*. *cholerae* also has a gap between these two elements, but it is much shorter than that of *leuO*, and TcpP is known to bind in this region where it binds ToxR and stimulates transcription^48^. We considered a similar mechanism for *leuO* regulation in *V. vulnificus*. However, an extensive search for a TcpP homolog in the genome of *V*. *vulnficus* strains was futile, leading us to consider the possibility that the bending of the DNA between the ToxR-binding site and the promoter was most relevant. The nucleotide sequences of the binding sites for ToxR and LeuO are AT rich, a property frequently associated with DNA bending mechanisms^49^. Furthermore, numerous LTTRs have been shown to induce DNA bending^26^. The number of bases between the ToxR binding site and the promoter of *leuO* is long enough to allow DNA bending to take place^50,51^. When we constructed two different deletion mutations in this region, we observed that a 5-bp deletion abolished expression of *leuO* but a 10-bp deletion had no effect. This supports the idea that an interaction between ToxR and RNA polymerase requires a bend in the DNA because a 5-bp deletion would result in a 180° rotation of the DNA helix, disrupting the interaction. It also is noteworthy that the *leuO* promoter sequence differs significantly from the canonical consensus sequence (Fig. 3), and therefore may require ToxR for binding of RNA polymerase.

The way in which LeuO represses its own expression is complex. The simplest model is one in which binding sites for LeuO overlap with both the ToxR binding site and the promoter sequence (LeuO-2 and LeuO-5 sites in Fig. 6a and Supplementary Fig. S4), and LeuO outcompetes RNA polymerase (Fig. 6d). Because LeuO binds to three additional sites, it is possible that binding to any of these sites leads to cooperative binding to the adjacent sites. If this is the case, we predict that deletions in any one of these sites would enhance expression of LeuO. However, in contrast to our expectations, 20-bp deletions in the middle of LeuO binding sites 1, 3, and 4 each led to a significant reduction in transcription of a *lacZ*-transcriptional fusion to *leuO* (Supplementary Fig. S5). Another possibility is that binding of LeuO to sites 1, 3, and 4 may in itself contribute to the bending of the DNA. Low levels of LeuO might stimulate bending and encourage transcription, but above a certain concentration, LeuO competes with ToxR or RNA polymerase, leading to an inhibition of transcription. Further physicochemical analysis of the interactions between the *leuO* upstream DNA, ToxR, RNA polymerase, and LeuO may answer these questions.

The physiological reasons for feedback inhibition of LeuO expression are unclear. Numerous genes are regulated by LeuO, including some with regulatory functions such as HU, AphA, and RpoS^13,21^. Continued high levels of expression of intracellular LeuO may intensify these downstream pathways and be harmful to the cell.

The nucleotide sequences of regions upstream of *leuO* in various *Vibrio* species are highly homologous (Supplementary Fig. S4). All are AT-rich and include putative LeuO binding sites in positions similar to those we have identified in *V. vulnificus*. Unlike *V. vulnificus*, *V. cholerae* has two ToxR binding sites in opposite orientations (ref. ^13^ and Supplementary Fig. S3), but the distance between these sites and the promoter is almost identical to that in *V. vulnificus*. The other species all appear to have a single ToxR binding site like *V. vulnificus*. This suggests that regulation of *leuO* is very similar among *Vibrio* species and that the cFP-ToxR-LeuO signaling circuit is well conserved, and may have evolved prior to the ToxT or TcpPH-dependent signaling proteins that are encoded on a pathogenic island unique to *V. cholerae*.

**Figure 7:**
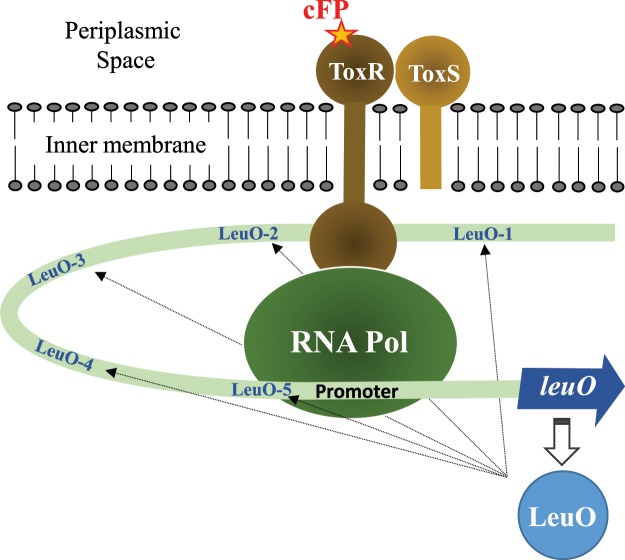
Working model of the combined *trans*- and *cis*-acting elements that regulate expression of *leuO*. When the cell receives a signal such as cFP, ToxR-signal transduction ensues. cFP binds to the periplasmic domain of ToxR, leading to a conformational change in the cytoplasmic domain of ToxR such that it binds to the ToxR box upstream of *leuO*. A curve in the region between the ToxR binding site and the promoter allows ToxR to directly interact with RNA polymerase and recruit it to the poor promoter sequence. High level of expression of LeuO leads to feedback inhibition of its own transcription, possibly through binding to the five sites in the upstream region and displacing ToxR and/or RNA polymerase. Alternatively, full occupation of the LeuO binding sites may interfere with DNA bending such that ToxR can no longer recruit RNA polymerase.

Numerous factors are involved in the regulation of *leuO* (summarized in a working model shown in Fig. 7). These *trans*-elements are individually controlled by their own signals and by other regulators. LeuO appears to play a crucial role in coordinating these complex combinations of signals to optimize the expression of numerous pathways for survival and pathogenicity. Because of this, LeuO may be a good candidate for the development of novel antibacterial agents to disrupt the normal physiology of the pathogen.

## Methods

### Strains, culture condition, and chemicals

Strains and plasmids used in this study are listed in Supplementary Table S1. All media were purchased from Difco (Detroit, MI) and all antibiotics were purchased from Sigma-Aldrich (St. Louis, MO). *Escherichia coli* strains were cultured in LB medium at 37 °C with appropriate antibiotics. *V. vulnificus* strains were cultured in LB medium or in thiosulfate citrate bile salt sucrose (TCBS) agar (Difco, Detroit, MI) at 30 °C. cFP (Bachem Inc, Switzerland) was dissolved in dimethyl sulfoxide (DMSO) and used at a final concentration of 5 mM.

### Cloning of *toxR* and construction of a *toxRS* deletion mutant derived from *V. vulnificus* MO6-24/O

All of the primers used in this study are listed in Supplementary Table S2. To clone *toxR*, a 1,111-bp DNA fragment comprising the promoter region and the coding region of *toxR* was amplified by PCR using primers toxR-comp-F and toxR-comp-R and cloned into the *Xho*I and *Kpn*I sites of pBBR1-MCS2^52^ to generate pBBR12-*toxR*. To construct a *toxRS* deletion mutant, a DNA fragment comprising the upstream region of *toxRS* was amplified using primers dtoxRS-up-F and dtoxRS-up-R and ligated to the pGEM-T Easy vector (Promega, Madison, WI) to generate pGEM-toxRSup. The downstream region of *toxRS* was amplified using primers dtoxRS-down-F and dtoxRS-down-R and ligated to the pGEM-T Easy vector to generate pGEM-toxRSdown. To construct a *toxRS* deletion, the *toxRS* upstream fragment from pGEM-toxRSup digested with *Xba*I and *Bam*HI and the *toxRS* downstream fragment from pGEM-toxRSdown digested with *Xho*I and *Bam*HI were cloned together into the pGEM-T Easy vector to construct pGEM-d*toxRS*. This resulting construct has a 1,401-bp deletion in *toxRS*. The plasmid pGEM-d*toxRS* was digested with *Xba*I and *Xho*I and ligated to pDM4^53^ to obtain pDM4-d*toxRS*, which was then introduced into *E. coli* S17-1 λ*pir*^54^ to be mobilized into *V. vulnificus* strain MO6-24/O by conjugation. Double crossover selection to construct a chromosomal deletion of *toxRS* was performed as described previously^53^. The deletion was confirmed by PCR and DNA sequencing, and the mutant was named MO6 *ΔtoxRS*.

### Cloning of *leuO* and construction of a *leuO* deletion mutation and a *toxRS*/*leuO* triple deletion in *V. vulnificus* MO6-24/O

For cloning of *leuO*, a 960-bp DNA fragment of the coding region of *leuO* was amplified by PCR using primers leuO-comp-F and leuO-comp-R. The resulting product was cloned into pBBR1-MCS2 to construct pBBR12-*leuO*. To construct a *leuO* deletion mutant, DNA fragments comprising the upstream and downstream regions of *leuO* were amplified using primers dleuO-F1 and dleuO-R1, and primers dleuO-F2 and dleuO-R2, respectively. The upstream region fragment was digested with *Bam*HI and the downstream region fragment was digested with *Bgl*II. The resulting DNA fragments containing the *leuO* upstream region and downstream region were ligated to the pGEM-T Easy vector to generate pGEM-d*leuO*. This resulting construct has a 492-bp deletion in the middle of *leuO*. Plasmid pGEM-d*leuO* was digested with *Sal*I and *Sph*I, and cloned into pDM4 to generate pDM4-d*leuO*, which was then introduced into *E. coli* S17-1 λ pir from which it was mobilized into either wild type *V. vulnificus* MO6-24/O or MO6-d*toxRS* by conjugation. Exconjugants were grown on LB agar containing chloramphenicol (2 μg/ml) to select for a double crossover as previously described^53^. The chromosomal *leuO* deletion mutations were confirmed by PCR and DNA sequencing, and the mutants were named MO6 *ΔleuO* and MO6 *ΔtoxRSΔleuO*, respectively.

### Construction of *lacZ*-transcriptional reporter fusions to *leuO*

A 540-bp and a 405-bp DNA fragment of the region upstream of *leuO* (−387 to +153 and −252 to +153 with respect to the translational start site, respectively) were amplified by PCR using primers lacZ-leuO-F and lacZ-leuO-R, and primers lacZ-sleuO-F and lacZ-leuO-R, respectively. The PCR products were cloned into pGEM-T Easy vector (Promega, Madison, WI) to construct pGEM-*leuO* and pGEM*-sleuO*. After verifying the sequences, the construct was digested with *Pst*I and *Bam*HI, and the fragments containing the upstream region of *leuO* were cloned into the *Pst*I and *Bam*HI sites of pRKΩ*lacZ*^2^. The resulting constructs were named pRK-*leuO*::*lacZ* and pRK-s*leuO*::*lacZ*, and mobilized into appropriate *V. vulnificus* strains via conjugation using *E. coli* strain S17-1.

A 540-bp DNA fragment of the region upstream of *leuO* (−387 to +153 with respect to the translational start site) was amplified by PCR using primers pMZtc-leuO-F and pMZtc-leuO -R. The PCR product was cloned into pGEM-T Easy vector (Promega, Madison, WI) to construct pGEM-pmZtc-*leuO*. After verifying the sequences, the construct was digested with *Xba*I and *Xho*I, and fragments containing the upstream region of *leuO* were cloned into the *Xba*I and *Xho*I site of pMZtc^21^. The resulting construct was named pMZtc-*leuO*. This construct was conjugated into *V. vulnificus* MO6-24/O wild type or appropriate strains, and a single crossover was obtained by selecting chloramphenicol-resistant colonies.

### β-Galactosidase assays

β–Galactosidase activity was measured as described previously^55^. Briefly, *V. vulnificus* strains were cultured overnight in LB medium and then washed and subcultured in fresh LB medium. To assess the effect of cFP, either 5 mM cFP or DMSO as a negative control was added, and samples were diluted to an A_600_ of 0.005.

### Purification of recombinant *LeuO* and western hybridization

Two primers, LysR-over2-F and LysR-over2-R, were used to amplify a 984-bp DNA fragment containing the complete open reading frame of *leuO* from the genomic DNA of *V. vulnificus*. *Nde*I and *Bam*HI sites located at the ends of the resulting *leuO* DNA were used for cloning into the pRE expression plasmid^43^ to generate plasmid pRE-LeuO. Recombinant LeuO was overexpressed in *E. coli* GI698 by adding tryptophan (final concentration 100 μg/ml) (Sigma) in M9 salt-based induction medium (0.2% casamino acids, 1% glycerol, and M9 salts) and purified using a Ni^+^-NTA affinity column as directed by the manufacturer (Qiagen). Purified LeuO was used to generate polyclonal antibodies through three consecutive immunizations of Sprague-Dawley rats (200 μg LeuO protein per immunization) at 3-week intervals. The polyclonal antibodies against LeuO were obtained as described previously^43^. For LeuO expression analysis, overnight cultures of *V. vulnificus* MO6-24/O, Δ*leuO*, or Δ*toxR* grown in LB were subcultured and, when necessary, treated with 5 mM cFP. Cells were collected when the A_600_ value of the culture reached approximately 0.6–0.8, and were resuspended in phosphate-buffered saline (PBS). Then, 50 μg of each lysate was subjected to SDS-PAGE and transferred to a Hybond P membrane (GE Healthcare Life Sciences, Piscataway, NJ). The membrane was incubated with polyclonal antibody against purified LeuO (1:2,000 dilution in blocking solution) and subsequently with goat anti-rat IgG-HRP (1:2000) (Santa Cruz Biotechnology, Santa Cruz, CA). LeuO expression was visualized using the NBT/BCIP color development substrate (Promega, Madison, WI) or the ECL western blotting detection reagent (GE Healthcare Life Sciences, Piscataway, NJ).

### Expression and purification of the cytoplasmic and periplasmic domains of ToxR

DNA fragments encoding 174 amino acids of the N-terminal cytoplasmic domain (named ToxR-N) and 98 amino acids of the C-terminal periplasmic domain (named ToxR-C) of ToxR were PCR-amplified using primers toxR-N-F and toxR-N-R and primers toxR-C-F and toxR-C-R, respectively. The amplified fragments were cloned into pET21a (Novagen, Madison, WI) using restriction enzymes *Sal*I and *Xho*I, to construct pET-21a::*toxR*-N and pET-21a::*toxR*-C, respectively. The resulting constructs encode ToxR-N and ToxR-C, each fused to a His-tag at the C-terminus. These plasmids were transformed into *E. coli* BL21 (DE3) (Novagen, Madison, WI), and expression was induced with 1 mM IPTG. After centrifugation, bacterial pellets were suspended in 1 × binding buffer (0.5 M NaCl, 5 mM imidazole, 20 mM Tris-HCl, pH 8.0) (Novagen, Madison, WI), sonicated, and centrifuged at 13,000 rpm for 10 min. The supernatant was applied to His·Bind resin (Novagen, Madison, WI) and bound proteins were eluted with 1 × elution buffer (1 M imidazole, 0.5 M NaCl, 20 mM Tris-HCl, pH 8.0). The purity of the eluted protein was confirmed by 15% SDS-PAGE.

### Isothermal titration calorimetry (ITC) for binding of cFP and the periplasmic domain of ToxR (ToxR-C)

The purified periplasmic domain of ToxR (ToxR-C) was used for the Isothermal Titration Calorimetry (ITC) analysis. ITC measurements were carried out using an automated ITC_200_ (MicroCal, GE Healthcare Life Sciences, Piscataway, NJ). The reference cell was filled with buffer (20 mM Na_2_HPO_4_ pH 7.8 and 1 mM EDTA). The reaction cell was filled with 0.2 mM of the periplasmic domain of ToxR, and a syringe was filled with either 10 mM cFP or 10 mM linear phenylalanine-proline dipeptide (FP). Two microliters of either cFP or FP was injected into the reaction cell containing ToxR-C at a concentration of 0.2 mM until saturated. Titrations were performed to give a series of 2 μl injections at 2 min intervals with 1,000 rpm stirring at 25 **°**C. As a control, cFP was titrated into the reference buffer (20 mM Na_2_HPO_4_ pH 7.8 and 1 mM EDTA). Data was evaluated in terms of a simple binding model using the ORIGIN software package (OriginLab, Northampton, MA).

### Expression and purification of ToxR

A DNA fragment encoding the entire 290 amino acids of ToxR was PCR-amplified using primers pASK-IBA3-toxR_F and pASK-IBA3-toxR_R. The amplified fragment was cloned into pASK-IBA3 vector, resulting in expression of ToxR fused to a strep-tag at the C-terminus. This plasmid was transformed into *E. coli* BL21(DE3) (Novagen, Madison, WI), and expression was induced with 0.2 μg/ml anhydrotetracycline. After harvesting the cells, the membrane protein ToxR was purified in the detergent *n*-dodecyl-β-D-maltopyranoside (DDM) as previously described with minor modifications^56,57^. Briefly, bacterial pellets were suspended in lysis/wash buffer (50 mM Tris-Cl, 150 mM NaCl, 1 mM EDTA, 2 mM dithiothreitol (DTT), 0.8% DDM, 10% glycerol, pH 8.0), sonicated, and then centrifuged at 13000 rpm for 20 min. The supernatant was applied to Strep-Tactin affinity resin (IBA Lifesciences, Göttingen, Germany), washed three times with lysis/wash buffer, and then specifically bound protein was eluted with buffer E (100 mM Tris-Cl, 150 mM NaCl, 1 mM EDTA, and 2.5 mM desthiobiotin). The eluted protein was loaded onto 12% SDS-PAGE to assess purity.

### Determination of the transcription start sites of *leuO* by primer extension

RNA was extracted from wild type *V. vulnificus* strain MO6-24/O grown in LB broth to an A_600_ of 1.0. The primer PE-leuO, which is complementary to the coding strand of *leuO*, was ^32^P-end labeled and used to synthesize cDNA from 1 μg of total RNA using PrimeScript RT Enzyme mix I (Takara Bio Inc., Japan). The same primer was used to generate a sequencing ladder using the Top DNA Sequencing Kit (Bioneer, Daejeon, Korea). The resulting primer extension product and sequencing ladders were resolved on a 6% polyacrylamide sequencing gel. The gel was dried and read using a BAS-1500 Imaging Plate (Fujifilm, Tokyo, Japan).

### Gel-mobility shift assays

To assess the binding of the ToxR to a *cis*-acting element of *leuO*, a 412-bp DNA fragment containing the upstream region of the *leuO* promoter (nucleotides +60 to −352 with respect to the translation start site of *leuO*) was PCR-amplified using leuO-F and leuO-R primers. To assess the binding of the ToxR-N to DNA fragment containing the region upstream of *leuO* including each of a 312-bp wild type fragment, the same fragments with a 5-bp or a 10-bp deletion between the promoter and the fragment with a deletion in the ToxR-binding site, and a 20-bp deletion in the ToxR binding site were amplified using primers lacZ-sleuO-F and leuO_R2. The PCR products were subsequently labeled with [γ-^32^P]ATP using T4 polynucleotide kinase (Enzynomics, Daejeon, Korea). For gel-mobility shift assays, 10 ng of the labeled DNA fragment was incubated with increasing amounts of purified ToxR (0 to 800 nM) or ToxR-N (0 and 100 nM) in a 20 μl reaction in binding buffer [10 mM Tris-HCl (pH 7.4), 10 mM KCl, 1 mM EDTA, 0.1 mM DTT, 50 μg/ml bovine serum albumin, and 5% glycerol] for 30 min at 30 °C. The reaction was terminated by the addition of 4 μl loading buffer, and samples were resolved on a 6% neutral polyacrylamide gel. The DNA was visualized using the BAS 1500 imaging system (Fujifilm, Tokyo, Japan).

### DNaseI footprinting assay

To determine the ToxR binding site on the upstream region of *leuO*, an end-labeled 266-bp DNA fragment of the *leuO* promoter region (nucleotides − 86 to − 352 relative to the translation initiation site) was amplified using primers leuO-F and ^32^P-labeled FP-leuO-R. To identify the LeuO binding site within the *leuO* promoter region, an end-labeled 244-bp DNA fragment of the *leuO* promoter region (nucleotides −143 to −387 relative to the translation initiation site), and an end-labeled 291-bp DNA fragment of the *leuO* promoter region (nucleotides +60 to −231 relative to the translation initiation site) were amplified using primers lacZ-leuO-F and ^32^P-labeled FP-leuO-R2, and leuO-F and ^32^P-labeled FP-leuO-R1, respectively. 200 ng of the amplified *leuO* promoter region was incubated with increasing amounts of purified ToxR and LeuO at 30 **°**C for 30 min in 50 μl of buffer (10 mM HEPES, 100 mM KCl, 200 μM EDTA, 10% glycerol, pH 7.5). After 30 min, 50 μl of CaCl_2_-MgCl_2_ solution (10 mM MgCl_2_, 5 mM CaCl_2_) was added to the reaction. Then, 0.25 U of DNase I (Promega, Madison, WI) was added and the reaction was incubated at room temperature for 1 min. These reactions were terminated through the addition of 90 μl stop solution (200 mM NaCl, 30 mM EDTA, 1% SDS). After addition of 500 μl ethanol, samples were precipitated on ice for more than 30 min and centrifuged. DNA pellets were washed with 70% ethanol, and resuspended in 10 μl loading buffer [0.1 M NaOH:formamide (1:2), 0.1% xylene cyanol, 0.1% bromophenol blue]. The samples and the sequencing ladder generated with ^32^P-labeled leuO-R were denatured for 5 min at 95 °C, chilled on ice, and loaded on to a 6% sequencing gel. The sequencing ladders were prepared using an AccuPower DNA sequencing kit (Bioneer, Daejeon, Korea). The DNA was visualized using the BAS 1500 imaging system (Fujifilm, Tokyo, Japan).

### ChIP analysis

ChIP analysis was performed following the manufacturer’s instructions (EZ ChIP, Upstate Biotechnology, Lake Placid, NY). Briefly, the *toxR* deletion mutant of *V. vulnificus* harboring pRK-toxR-strep-tag was grown overnight in LB broth, then subcultured to an A_600_ of 0.005 in fresh LB broth containing either 5 mM cFP or DMSO. When the culture reached an A_600_ of 2.0, cells were treated with 1% formaldehyde and incubated at room temperature (RT) for 15 minutes. After incubation, 0.125 M glycine was added to quench unreacted formaldehyde, then incubated for 5 min at room temperature. Cells were harvested and washed with cold phosphate-buffered saline (PBS). Concentrated cells were sonicated, and cell lysates were incubated with Protein G Agarose beads (Santa Cruz Biotechnology, Santa Cruz, CA) in dilution buffer for 1 hour at 4 **°**C. Protein G agarose beads were collected by centrifugation, supernatants were removed, and pellets were treated with either Anti Strep-tag II monoclonal antibody (IBA Lifesciences, Göttingen, Germany) or normal mouse IgG (Santa Cruz Biotechnology, Santa Cruz, CA). After incubation at 4 **°**C overnight, protein G agarose beads were added and further incubated for 1 hour at 4 °C. Protein G agarose beads were pelleted and then washed with low salt immune complex buffer, high salt immune complex wash buffer, LiCl immune complex wash buffer, and TE buffer, sequentially. Washed agarose beads were treated with elution buffer, and then the protein/DNA complex was reverse cross-linked by incubation at 65 **°**C for 5 hrs. Free DNA was purified using a DNA purification kit (Biofact, Deajeon, Korea) following the manufacturer’s directions. DNA was analyzed by quantitative real-time PCR (qRT-PCR) on a Light Cycler 480 II real-time PCR system (Roche Applied Science, Upper Bavaria, Germany). qRT-PCR was carried out in triplicate in a 96-well plate (Roche Applied Science) using the primers in Supplementary Table S2. The gene encoding NAD-dependent glyceraldehyde-3-phosphatase of *Vibrio* species was used as an endogenous loading control for the reactions. Quantification was carried out using the Light Cycler 480 II real-time PCR system software program.

### Construction of pBAD-toxRS

A 1431-bp fragment of *toxRS* was amplified by PCR using primers pBAD_toxRS_F and pBAD_toxRS_R using the genomic DNA of *V. vulnificus* as a template. The resulting product was cloned into the pBAD-TOPO (Invitrogen, Carlsbad, CA) vector to generate pBAD-toxRS.

### *In vitro* pull-down assay to assess the binding of RNA polymerase to the LeuO promoter

DNA fragments containing the region upstream of *leuO* including each of a 5-bp and a 10-bp deletion between the promoter and the ToxR-binding site, and a 20-bp deletion in the ToxR binding site were amplified using primers lacZ-leuO-F and leuO_pull down_R. 50 nM of each DNA fragment was incubated with 100 nM of purified ToxR-N, 50 nM of RNA polymerase holoenzyme (New England Biolabs, Beverly, MA), and LeuO (0 to 200 nM) in a 30 μl reaction in binding buffer [10 mM Tris-HCl (pH 7.4), 10 mM KCl, 1 mM EDTA, 0.1 mM DTT, 50 μg/ml bovine serum albumin, and 5% glycerol] for 1 hour at 30 °C. The reaction was applied to Streptavidin Magnetic Beads (New England Biolabs, Beverly, MA), and then incubated for 4 hours at room temperature. The streptavidin magnetic beads were washed twice with wash buffer [12 mM HEPES-NaOH (pH 7.9), 4 mM Tris-Cl, 30 mM KCl, 1 mM EDTA, 1 mM DTT, and 12% glycerol]. Washed resins were treated with 30 μl of protein loading buffer, and samples were boiled for 5 min. The samples were centrifuged to pellet the streptavidin magnetic beads and SDS-PAGE was used to analyze the supernatant. After transfer to a Hybond P membrane (GE Healthcare Life Sciences, Piscataway, NJ), the membrane was incubated with anti-*E. coli* RNA Polymerase alpha monoclonal antibody (1:2,000 dilution in blocking solution) (Biolegend, San Diego, CA) and subsequently with goat anti-mouse IgG-HRP (1:2000) (Santa Cruz Biotechnology, Santa Cruz, CA). The amount of RNA polymerase alpha subunit was visualized using ECL Western blotting detection reagent (GE Healthcare Life Sciences, Piscataway, NJ).

### Ethical approval and informed consents

This study was carried out in accordance with the recommendations in the Animal Protection Act of Korea. The protocol was approved by the Institutional Animal Care and Use Commit Sogang University (Approval number: IACUCSGU2019_07). Rats were consistently monitored for signs of distress over the course of the experiments to be removed from the experiment and euthanized using carbon dioxide inhalation to prevent unnecessary suffering.

## Supplementary information

Supplementary Information.

## Data Availability

Data and associated protocols are promptly available to readers as requested.
