# Supplementary material for: Multi-Factor Regulation of the Master Modulator LeuO for the Cyclic-(Phe-Pro) Signaling Pathway in Vibrio vulnificus
Source: Sci Rep. 2019 Dec 27;9:20135. doi: 10.1038/s41598-019-56855-4 (PMC6934829; doi:10.1038/s41598-019-56855-4)

Supplementary Information

Multi-Factor Regulation of the Master Modulator LeuO for the Cyclic-(Phe-Pro) Signaling Pathway in *Vibrio vulnificus*

Na-Young Park[1], In Hwang Kim[1], Yancheng Wen[1], Keun-Woo Lee[1], Sora Lee[1], Jeong-A Kim[1], Kwang-Hwan Jung[1,3], Kyu-Ho Lee[1], and Kun-Soo Kim[1,2]

Department of Life Science[1] and Interdisciplinary Program of Integrated Biotechnology[2], and Institute of Biological Interfaces[3], Sogang University, Seoul, Korea.

Table S1. Bacterial strains and plasmids used in this study.

Strains or plasmids	Genotypes	Sources
**Strains**		
E. coli		
DH5α	λ^-φ80d*lacZ*Δ*M15*Δ(*lacZYA*-*argF*)*U169* *recA1* *endA1* *hsdR17* (r_K^- m_K^-) *supE44* *thi-1* *gyrA* *relA1*	Our collection
S17-1	[C600::RP4-2 (Tc::Mu)(Km::Tn7) *thi pro hsdRM*$^+$ *recA*	1
S17-1 λ *pir*	S17-1 with λ*pir* lysogen	1
BL21(DE3)	F^- *ompT hsdSB* (r_B^-,m_B^-) *gal dcm* (DE3)	Novagen
GI698	F− λ− *lacI*q *lacPL*8 *ampC*::Ptrp cI	2
V. vulnificus		
MO6-24/O	Pathogenic clinical isolate	3
MO6 Δ*toxR*	Derivative of MO6-24/O with a deletion in *toxR*	4
MO6 Δ*toxRS*	Derivative of MO6-24/O with a deletion in *toxRS*	This study
MO6 Δ*leuO*	Derivative of MO6-24/O with a deletion in *leuO*	This study
MO6 Δ*toxRS*Δ*leuO*	Derivative of MO6-24/O with double deletion in *toxRS* and *leuO*	This study
**Plasmids**		
pDM4	Suicide vector for allelic exchange, *sacB*, Cm^r	5
pDM4-d*toxRS*	pDM4 with the deletion of *toxRS* of *V. vulnificus*	This study
pDM4-d*leuO*	pDM4 with the deletion of *leuO* of *V. vulnificus*	This study
pRKΩ*lacZ*	Promoterless *lacZ* vector	4
pRK-*leuO*::*lacZ*	pRKΩ*lacZ* vector with the *leuO* upstream region (-387 to +153)	This study
pRK-s*leuO*::*lacZ*	pRKΩ*lacZ* vector with the *leuO* upstream region	This study

	(-252 to +153)	
pRK-s*leuOΔ5bp*::*lacZ*	pRKΩ*lacZ* vector with the 5-bp (-177 to -173) deletion mutated region in *leuO* upstream region (-252 to +153)	This study
pRK-s*leuOΔ10bp*::*lacZ*	pRKΩ*lacZ* vector with the 10-bp (-177 to -168) deletion mutated region in *leuO* upstream region (-252 to +153)	This study
pRK-s*leuOΔtoxR*::*lacZ*	pRKΩ*lacZ* vector with the deletion mutated region in ToxR binding site of the *leuO* upstream region (-252 to +153)	This study
pMZtc	pDM4 with the promoter-less *lacZ* gene for transcriptional fusion	6
pMZtc-*leuO*	pMZtc with the promoter region of *leuO*	This study
pRE1-*leuO*	*leuO* expression vector	7
pET21a	Expression vector, C-terminal His-tag, Ap^r	Novagen
pET-ToxR-N	pET21a vector with *V. vulnificus* N-terminal domain of ToxR	This study
pET-ToxR-C	pET21a vector with *V.vulnificus* C-terminal domain of ToxR	This study
pASK-IBA3	Expression vector, C-terminal strep-tag, Ap^r	IBA
pASK-ToxR	pASK-IBA3 vector with *V.vulnificus* ToxR	This study
pBBR1-MCS2	Broad host range expression vector, Km^r	8
pBBR12-*toxR*	pBBR1-MCS2 with *V. vulnificus toxR*	This study
pBBR12-*leuO*	pBBR1-MCS2 with *V. vulnificus leuO*	This study
pBBR12-*leuO*-ara	pBBR-MCS2 vector with the *V. vulnificus leuO* under the *araC* promoter	6
pBAD-TOPO	Arabinose regulated expression plasmid, Ap^r	Invitrogen
pBAD-ToxRS	pBAD-TOPO vector with *V.vulnificus* ToxRS	This study

* Numbers indicate nucleotide positions relative to the translational start site

1 Simon, R., Priefer, U., & Pühler, A. A broad host range mobilization system for *in vivo* genetic engineering: transposon mutagenesis in gram negative bacteria. *Nature*

Biotechnol. **1**, 784-791 (1983).

2 Lavallie, E. R. *et al.* A thioredoxin gene fusion expression system that circumvents inclusion body formation in the *E. coli* cytoplasm. *Nature Biotechnol.* **11**, 187 (1993).

3 Reddy, G. P. *et al.* Purification and determination of the structure of capsular polysaccharide of *Vibrio vulnificus* M06-24. *J. Bacteriol.* **174**, 2620-2630 (1992).

4 Park, D. K. *et al.* Cyclo(Phe-Pro) modulates the expression of *ompU* in *Vibrio* spp. *J. Bacteriol.* **188**, 2214-2221, https://doi.org/10.1128/JB.188.6.2214-2221.2006 (2006).

5 Milton, D. L., O'Toole, R., Horstedt, P. & Wolf-Watz, H. Flagellin A is essential for the virulence of *Vibrio anguillarum*. *J. Bacteriol.* **178**, 1310-1319 (1996).

6 Kim, I. H. *et al.* Cyclo-(l-Phe-l-Pro), a quorum-sensing signal of *Vibrio vulnificus*, induces expression of hydroperoxidase through a ToxR-LeuO-HU-RpoS signaling pathway to confer resistance against oxidative stress. *Infect. Immun.* **86**, https://doi.org/10.1128/IAI.00932-17 (2018).

7 Kim, J. A. *et al.* Stationary-phase induction of *vvpS* expression by three transcription factors: repression by LeuO and activation by SmcR and CRP. *Mol. Microbiol.* **97**, 330-346, https://doi.org/10.1111/mmi.13028 (2015).

8 Kovach, M. E. *et al.* Four new derivatives of the broad-host-range cloning vector pBBR1MCS, carrying different antibiotic-resistance cassettes. *Gene.* **166**, 175-176 (1995).

Supplementary Table S2. Primers used in this study.

Function and Name	Nucleotide sequence (5’ → 3’)
**Cloning of *toxR* and construction of *toxRS* deletions**	
toxR-comp-F	CTCGAGGTTCATTTTCACTCCAAAG
toxR-comp-R	GGTACCGTGATTATTTACAGATAG
dtoxRS-up-F	TCTAGACTTCACGCATGTGCA
dtoxRS-up-R	GGATCCTGAGGTTCTTCTTTATAT
dtoxRS-down-F	GGATCCGATGCTCGTTTAGTCAT
dtoxRS-down-R	CTCGAGATCCATGCGCCAAGT
**Cloning of *leuO* and construction of *leuO*-deletion mutation and *toxRS/leuO* double mutations**	
leuO-comp-F	CTCGAGATGTTAGATAAAAAA
leuO-comp-R	TCTAGATTATACCGCAGCAAC
dleuO-F1	TTGAGCAACAAAGCAACGT
dleuO-R1	ATGGATCCATTCGATAGCTGGCAAT
dleuO-F2	AGGCTTCTCAAGCACAGAG
dleuO-R2	CAAAATCGGCCACCGTCCAC
**Construction of *leuO* transcriptional fusion**	
lacZ-leuO-F	CTGCAGAAGTCGACTTTCCCA
lacZ-leuO-R	GGATCCCTGTGACATGCCTAA
lacZ-sleuO-F	CTGCAGGCTACTTATGAGGGT
PMZtc-leuO-F	CTCGAGAAGTCGACTTTCCCAGTTACGTG
pMZtc-leuO-R	TCTAGACTGTGACATGCCTA

Expression of recombinant LeuO

LysR-over2-F	GGAATTCCATATGATGTTAGATAAAAAAGATGCGATGAGCGCG
LysR-over2-R	CGGGATCCGTAACAGGCCGTGTCGGTTATACCGC

Purification of recombinant ToxR

toxR-N-F	GTCGACTCATGAGTAATATCGGC
toxR-N-R	CTCGAGATTTTTTTGTGACGCGGC
toxR-C-F	GTCGACTTATGTTGCTCACCAAT
toxR-C-R	CTCGAGTTTACAGATAGAGCCAAG
pASK-IBA3-toxR-F	GAATTCATGAGTAATATCGGCAC
pASK-IBA3-toxR-R	CTCGAGTTTACAGATAGAGCCAAG

Construction of pBAD-toxRS

pBAD-toxRS-F	ATGAGTAATATCGGCACTAAGTTTG
pBAD-toxRS-R	TCCCTTATTTGATGACTAAAC

Primer extension, gel mobility shift assay, and DNaseI footprinting

PE-leuO	GCGTAAAGTACTTTCCATTCGA
leuO-F	GGTACCCGCTAATATTGACGA
leuO-R	TCTAGATAAAGTACTTTCCATTC
leuO-R2	TCTAGATAAAGTACTTTCCATTCG
FP-leuO-F	GGTACCGTTTTTTATAGTTTTTTAG
FP-leuO-R1	ATCACTCTCAGGGCGCAGCTCA
FP-leuO-R2	TCTAGATTGATAATTAATCACA
FP-leuO-R3	TAAAGTACTTTCCATTCGATAGC

**ChIP analysis**	
leuO-chIP-F	CGGAGTGGATCTCAACCTACTGAC
leuO-chIP-R	ACGCATGAAAAGCTCATCATTAAA
ompU-chIP-F	TGACCGTGCAGATTCAAGCAAGA
ompU-chIP-R	AACCACCACCAAGCGTTAGACCA
GAPDH-F	CGT ATC GGT CGT TTC GTT TT
GAPDH-R	TAC GTC AAC ACC GAT TGC AT
***In vitro* pull down assay**	
leuO-pull down-R	Biotin-GTACTTTCCATTCGATAGCTG

Nucleotides modified for the generation of restriction sites is underlined.

Supplementary Figure S1. The region upstream of *leuO*is bound by LeuO.

DNaseI protection assay of the region upstream of *leuO* using purified LeuO. Left panel: 200 ng of labeled *leuO* promoter DNA was combined with either no LeuO, lanes 1 and 6, or 0.5 μM, 0.8 μM, 1 μM, and 2 μM LeuO, lanes 2-5, respectively. Right panel: 200 ng of labeled *leuO* promoter DNA was combined with either no LeuO, lanes 1 and 5, or 0.5 μM, 0.8 μM, and 1 μM LeuO, lanes 2-4, respectively. Regions protected by LeuO and the nucleotide sequence of each are indicated to the right.

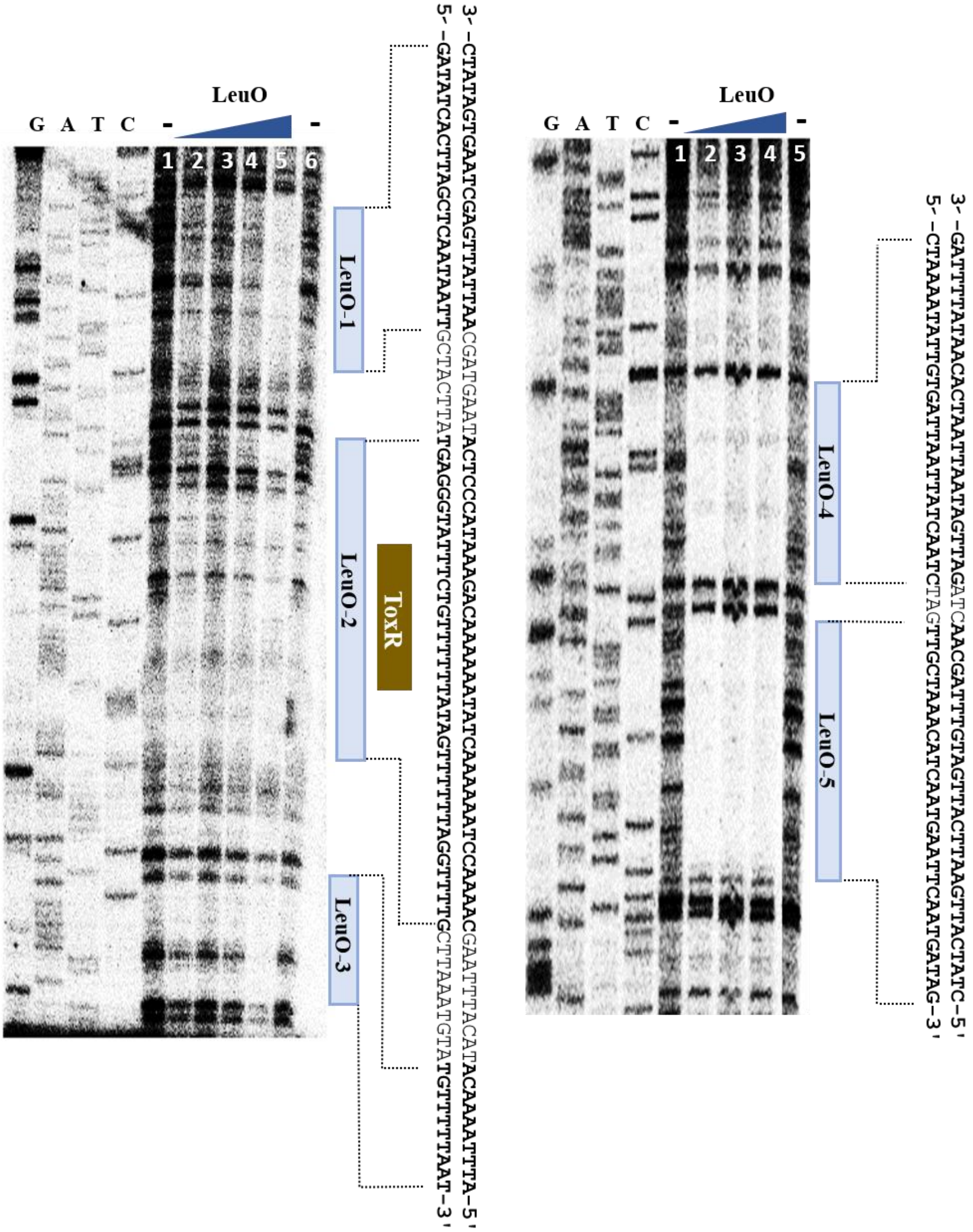

Supplementary Figure S2. A consensus sequence of LeuO binding site.

Alignment of the 19-nucleotide sequence common to all five LeuO binding sites identified in this study to the previously reported LeuO-binding sequences from *V. vulnificus* and *E. coli*[46]. Nucleotide sequences were aligned using CLC sequence viewer. Red, orange, yellow, and green shading indicates approximately 100%, 75%, 50%, and less than 20% identity, respectively. The illustration of this consensus binding motif was compiled by the WebLogo tool (http://weblogo.berkeley.edu/).

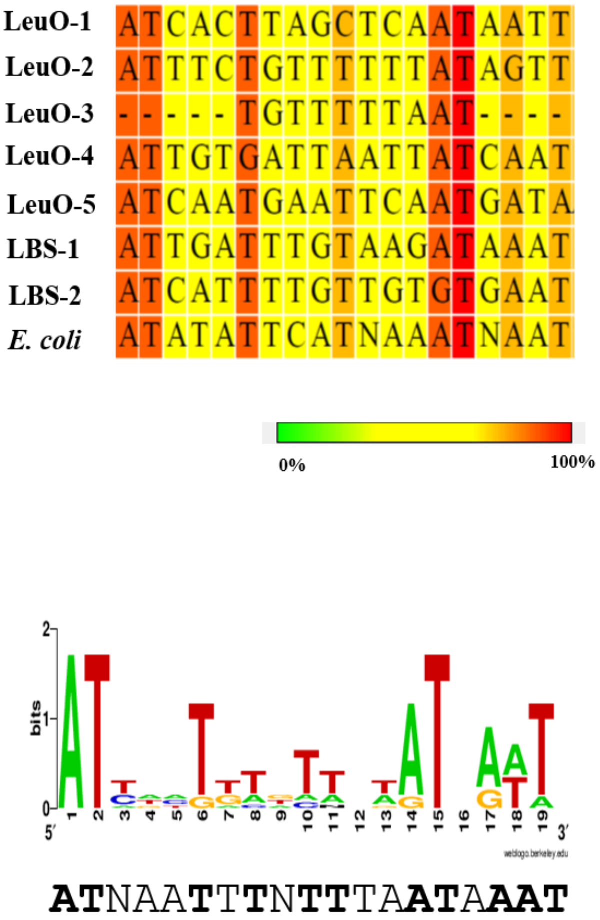

Supplementary Figure S3. A comparison of the upstream DNA regions of genes in *V. cholerae* regulated by ToxR.

For each of four DNA sequences, promoter regions are indicated by green arrows and consensus ToxR binding sites are indicated by yellow arrows. The upstream region of *leuO* has only one ToxR binding site (dotted yellow arrow) in *V. vulnificus*, but two inverted sites in *V. cholerae* (solid yellow arrows). The TcpP binding site is indicated with a pink box. The location of each site relative to the start of transcription is indicated [Crawford, J. A., Kaper, J. B. & DiRita, V. J. Analysis of ToxR-dependent transcription activation of *ompU*, the gene encoding a major envelope protein in *Vibrio cholerae*. *Mol. Microbiol.***29**, 235-246 (1998)].

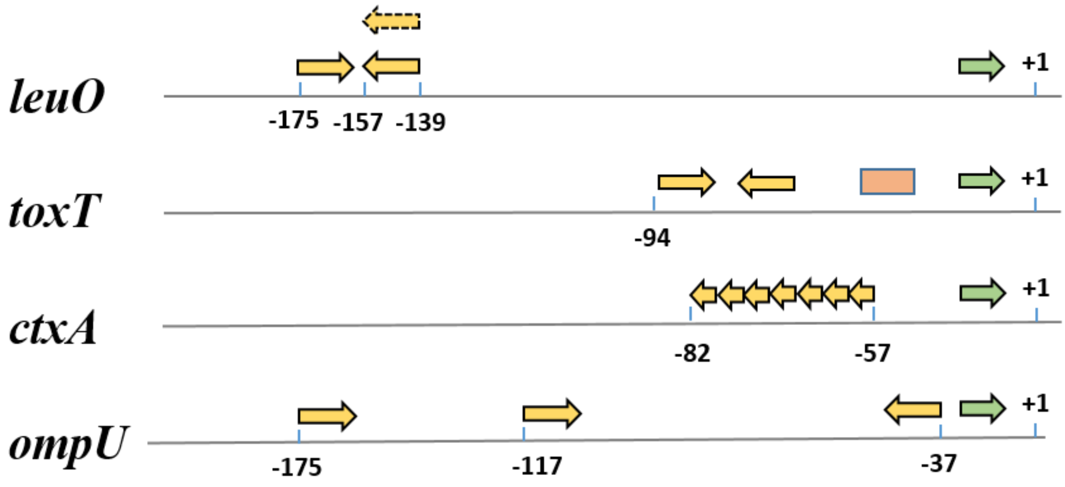

Supplementary Figure S4. DNA sequence alignment comparing regions upstream of *leuO* in *Vibrio* species.

Nucleotide sequences were aligned using CLC sequence viewer. Red, orange, yellow, and green shading indicates approximately 100%, 75%, 50%, and less than 20% identity, respectively. The transcription start site (+1) and promoter regions (-35 and -10) are noted. Binding sites for ToxR, LeuO are indicated. Strains from which these *leuO* sequences originated and the associated NCBI accession numbers are as follows: *V. vulnificus* MO6-24/O (VVM06_02645), *V. cholerae* El Tor N16961 (VC2485), *V. parahaemolyticus* RIMD 2210633 (VP0350), *V. harveyi* ATCC 43516 (AL538_RS12030), *V. navarrensis*ATCC 51183 (EA26_RS02630), *V. cidicii* 2756-81 (AUQ44_RS10800), *V. alginolyticus* K10K4 (K10K4_RS01815), *V. diabolicus* FDAARGOS_105 (AL537_06395), *V. rotiferianus* B64D1 (BSZ04_RS21800).

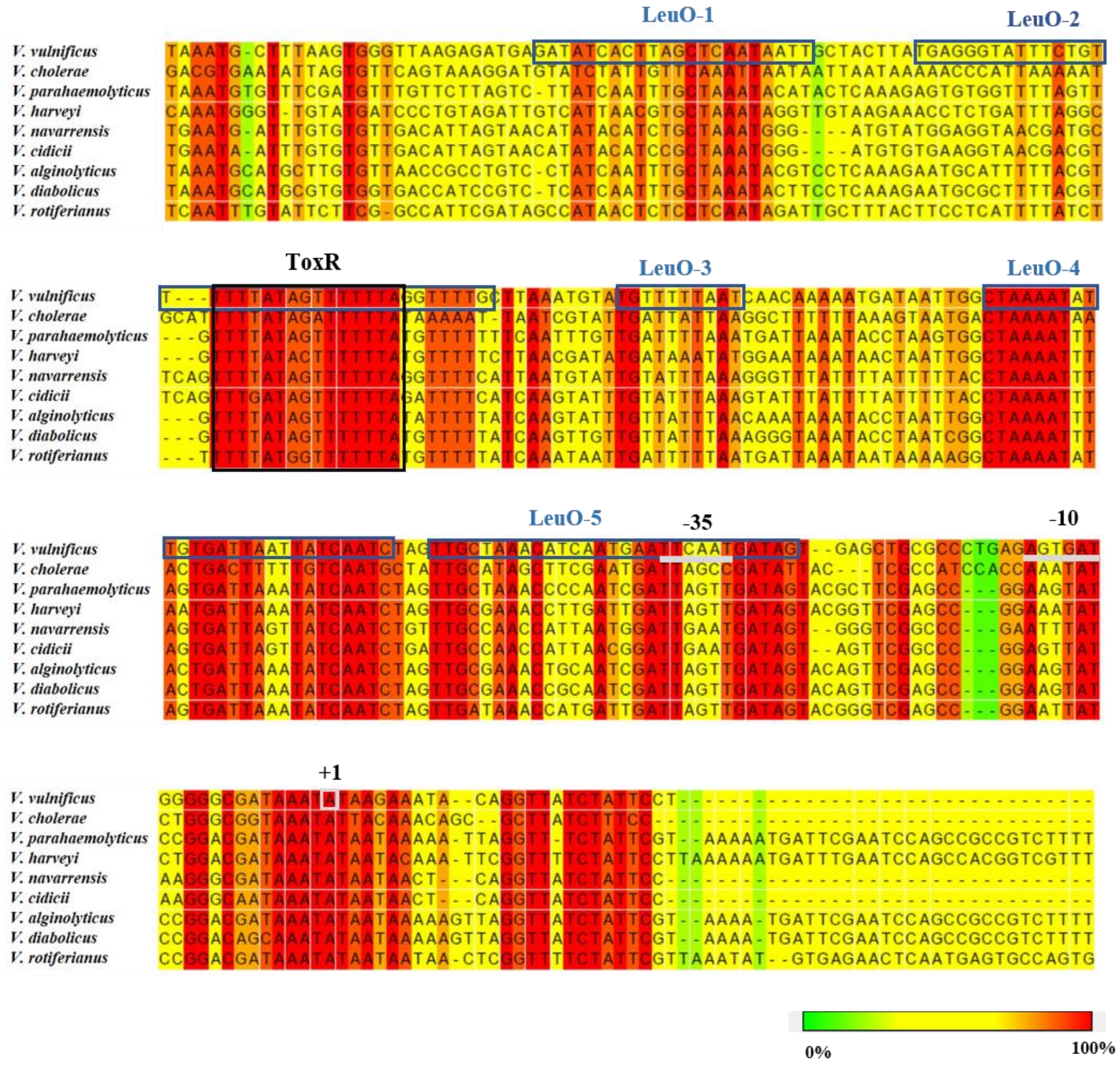

Supplementary Figure S5. Effect of deletions in LeuO binding sites upstream to *leuO* on the expression of *leuO*.

β-Galactosidase activities of *leuO*-*lacZ* fusions to a series of deletions in the upstream region of *leuO* in the absence and presence of exogenous 5 mM cFP in wild type *V. vulnificus*.

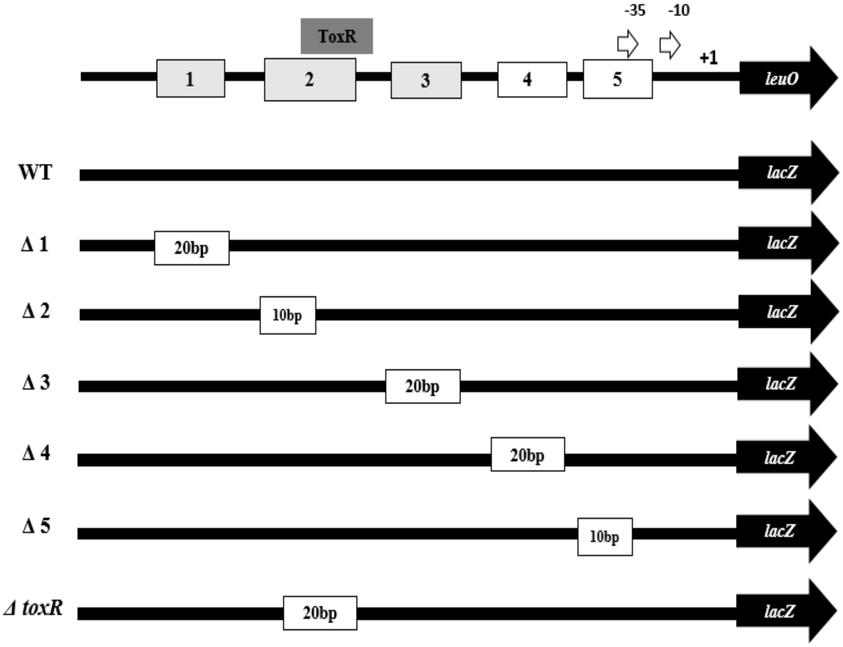

Relative transcription level

	cFP-	cFP+
WT	1 ± 0.09	2.3 ± 0.07
Δ 1	0.63 ± 0.08	1.62 ± 0.03
Δ 2	0.49 ± 0.02	0.81 ± 0.1
Δ 3	0.77 ± 0.04	1.14 ± 0.02
Δ 4	0.18±0.03	0.18±0.05
Δ 5	0.14±0.01	0.15±0.03
Δ *toxR*	0.16±0.03	0.15±0.04

Supplementary Figure S6.
The original image for Figure 1b.

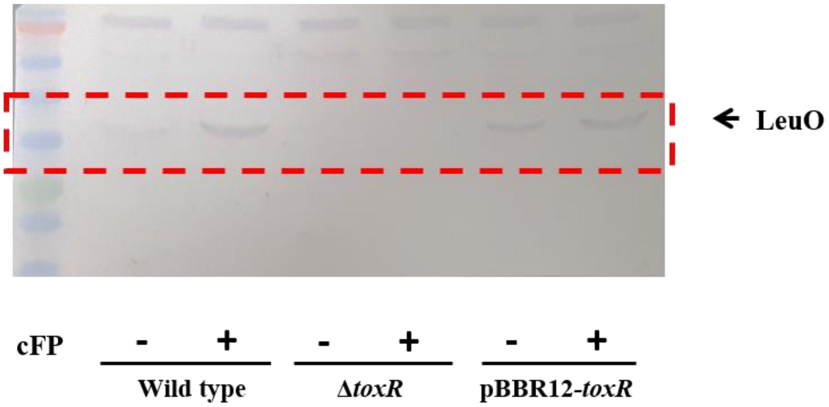

Supplementary Figure S7.
The original images for Figure 3a at three different exposures

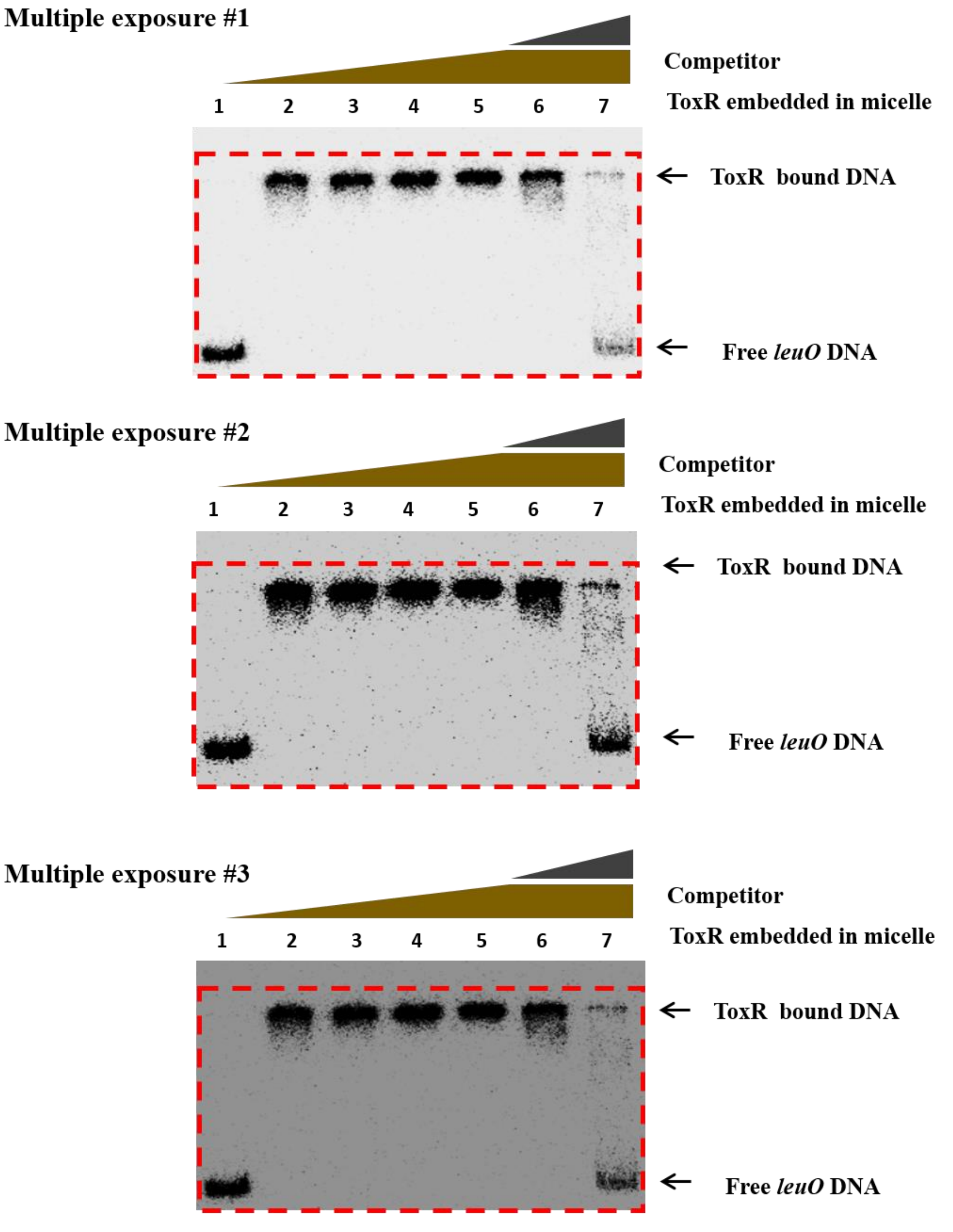

Supplementary Figure S8.
The original image for Figure 5b.

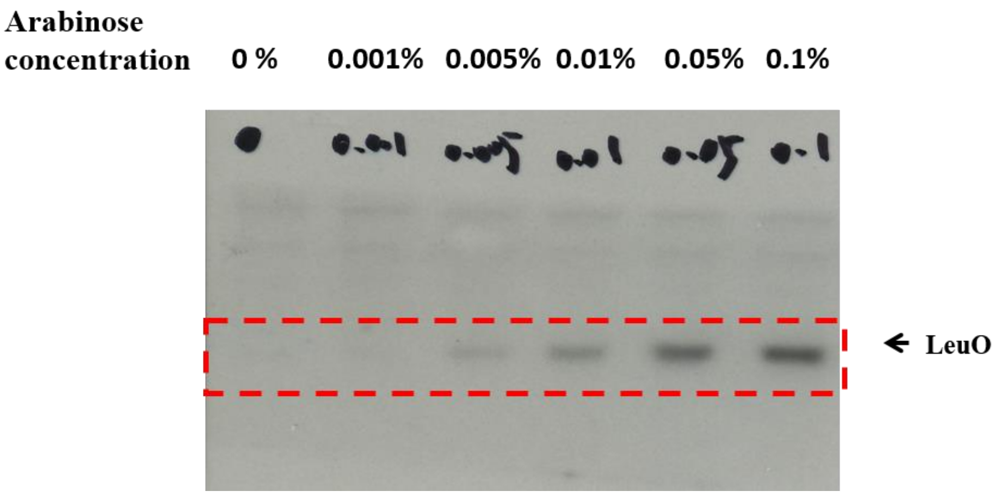

Supplementary Figure S9.
The original images for Figures 6c and d.

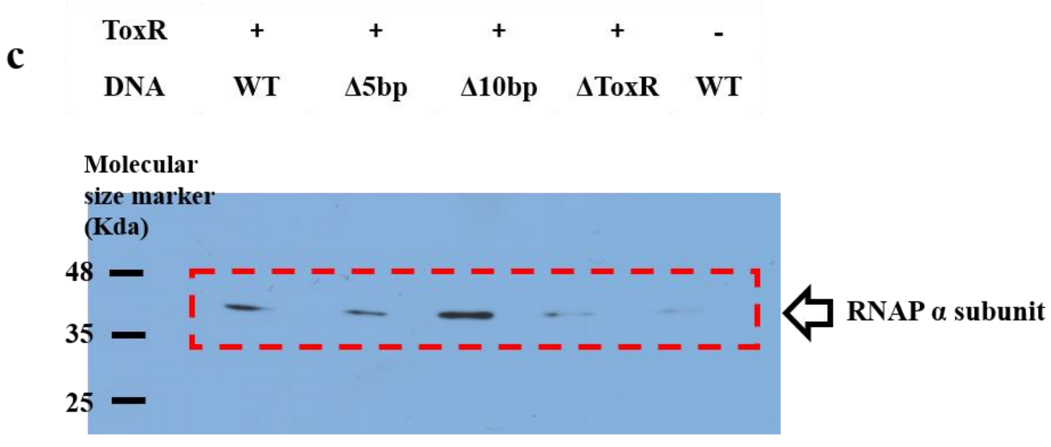

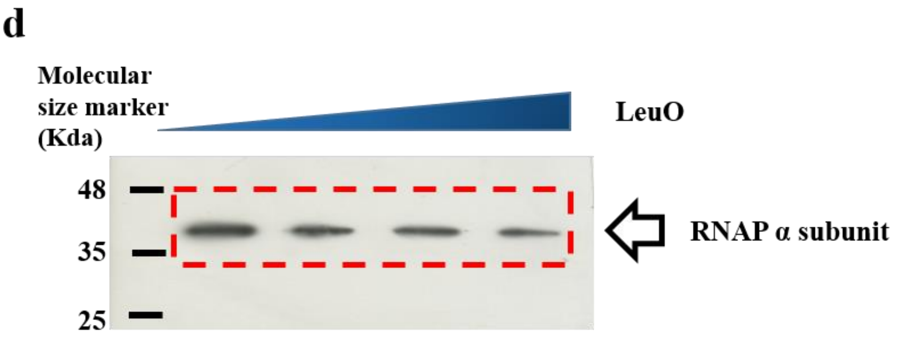

Supplementary Figure S10.
The original images for Figure 6e at three different exposures.

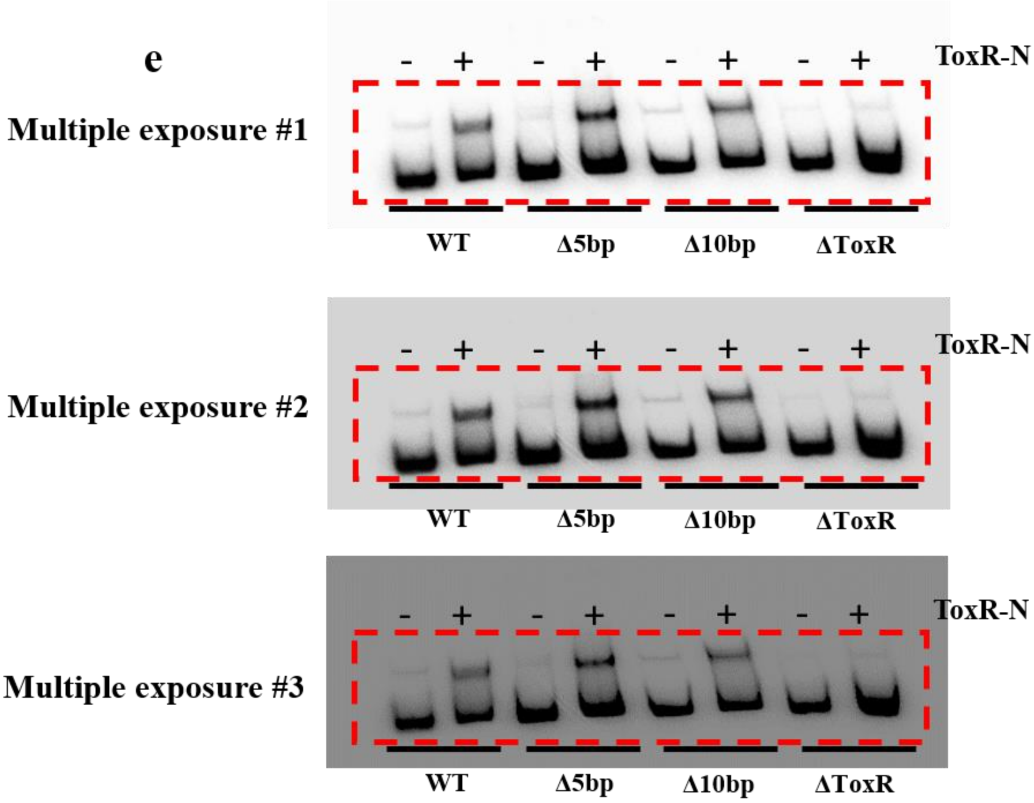

Supplement: Supplementary file 1 — Supplementary Information. [file 41598_2019_56855_MOESM1_ESM.pdf]
